# Broadening the vaccine metaphor: The adequate balanced food (ABF) vaccine against tuberculosis (Acid-fast bacilli/AFB) and more

**DOI:** 10.1371/journal.pgph.0004719

**Published:** 2025-06-24

**Authors:** Anurag Bhargava

**Affiliations:** 1 Department of Medicine, Kasturba Medical College Mangalore, Manipal Academy of Higher Education, Manipal, India; 2 Department of Medicine, McGill University, Montreal, Canada; ICMR-National Institute for Research in Tuberculosis: National Institute of Research in Tuberculosis, INDIA

## Abstract

Nutrition is essential to survival, health, and protection from disease across the lifespan. In the 1970s, an adequate diet was described as the most effective vaccine available for respiratory, diarrheal, and other common infections, as nutritional supplementation reduced these in the setting of undernutrition. Recently, the RATIONS (Reducing Activation of Tuberculosis through Improvement Of Nutritional Status) trial showed the efficacy of nutritional supplementation in reducing TB incidence in households by up to 50%, and an editorial used the metaphor of food as a vaccine for tuberculosis. This essay provides a historical overview of nutrition and TB prevention, with reports of reduced TB incidence from nutritional supplementation in World War II prisoner-of-war camps. This essay discusses additional evidence supporting McKeown’s proposition that the historical decline of TB in countries like the UK was related to improvements in nutrition. Undernutrition is the leading risk for tuberculosis incidence globally, the underlying cause of 45% of 4.9 million deaths in children under five years annually. Undernutrition in early life is a risk factor for many non-communicable diseases, and its effect on cognition and growth perpetuates both undernutrition and poverty intergenerationally. The essay broadens the vaccine metaphor to describe adequate balanced food (ABF) as a vaccine for TB and many public health problems, with a unique product profile. It concludes with a reminder that nutrition acts by optimizing immune function - the most powerful system/vaccine we have for TB prevention; draws attention to emerging threats to food security like climate change and conflicts, and proposes that the answer to the prevention of TB may lie in better population health rather than only a war on the bacillus.

## Introduction

“The control of infectious diseases by specific measures such as vaccination, or general action such as environmental improvement, has a favourable impact on a community’s nutritional status. On the other hand, adequate food offers good protection against the more serious effects of communicable diseases, including even those against which we still have no accurate or easy measures. For the time being, an adequate diet is the most effective “vaccine” against most diarrhoeal, respiratory, and other common infections.”

-Dr. Moisés Béhar, Director of the Institute of Nutrition of Central America and Panama, Guatemala, and later Director of Nutrition at WHO [1].

Nutrition is essential to survival, health, and protection from disease across the lifespan. The Merriam-Webster medical dictionary defines a vaccine as “a preparation that is administered (as by injection) to stimulate the body’s immune response against a specific infectious agent or disease.” [2] Nearly 50 years after Dr. Moisés Béhar used the vaccine metaphor for an adequate diet, we published the results of the first field-based cluster-randomized trial of nutritional supplementation to reduce TB incidence in household contacts (HHCs) of patients with microbiologically confirmed pulmonary tuberculosis [3]. The editorial on the RATIONS (Reducing Activation of Tuberculosis through Improvements Of Nutritional Status) trial was titled, “Food the tuberculosis vaccine we already have.” [4] In the trial, food baskets and multivitamins given for 6 months reduced the rates of incident TB in HHCs over the next 2 years by 39% for all forms of TB and nearly 48% in the case of lung TB [3]. In a sensitivity analysis, the reduction in hazard of incident TB of all forms and of lung TB in the RATIONS trial was 41% and 49%, respectively [3]. The effects of the nutritional supplementation were similar to the protection against lung TB (49.7%) seen in the phase 2b trial of the promising candidate - M 72/ASO1E vaccine [5]. The final results of the phase 3 trial are expected by 2028.

This essay provides a historical overview of nutrition and TB prevention. It then describes adequate balanced food as a vaccine against tuberculosis and much more, with a unique product profile with 10 outstanding features. The essay concludes with a reminder that nutrition acts by optimizing the function of the most powerful system/vaccine we have for TB prevention. It proposes that the answer to the prevention of TB may lie in better population health rather than only a war on the bacillus.

## Nutrition in TB prevention

### Nutrition and reduction of TB incidence from natural experiments in the early 20^th^ century

The protection observed with a nutritional intervention in the RATIONS trial was neither novel nor exceptional. The protective effect of nutrition on TB incidence, with even higher reductions in TB incidence, has been demonstrated in some well-documented natural experiments in the first half of the 20th century. At the Papworth village settlement, adequate nutrition was considered the main social intervention (apart from improved housing and stable employment) for an 84% reduction in TB incidence in HHCs of patients with active tuberculosis (PwTB), which was not accompanied by any decrease in transmission of TB infection [6]. In prisoners of war (POW) camps in Germany, while British and Russian soldiers lived in similar conditions of stress and overcrowding, only 1.2% of British soldiers developed TB compared to 15% of Russian soldiers in one such camp [7]. This 92% reduction in TB incidence was related to the additional Red Cross ration parcel available only to British soldiers. While the Russians subsisted only on the camp diet, the British had an additional 1000 KCal and 30 g of protein daily. Archibald Cochrane, a British doctor and later the founder of the Cochrane Collaboration, corroborated this observation at another POW camp where he served [8]. In a population that was screened for TB, the incidence of TB was 6% among Russians, 0.5% among the French, and 0% among the British. Archie Cochrane wrote, “Comment on the high incidence of tuberculosis among the Russians is almost superfluous. The raised incidence among the French in 1945 was doubtless a reflection of the absence of parcels from France after 1944.” [8]

### Revisiting in the END TB era the historical decline in TB incidence and TB mortality through a nutritional lens

The world has seen a net percentage decline of 8.3% in TB incidence and 23% in TB mortality between 2015–2023, far from the 50% decline in TB incidence and 75% decline in TB mortality desired by 2025 [9]. The current strategy admits that a more holistic approach combining biomedical, public health, and socioeconomic interventions will be required to achieve the desired targets [10]. Here, some historical facts on TB morbidity and mortality trends in the pre-chemotherapy and pre-BCG era have relevance [11–13].

**Fig 1:**
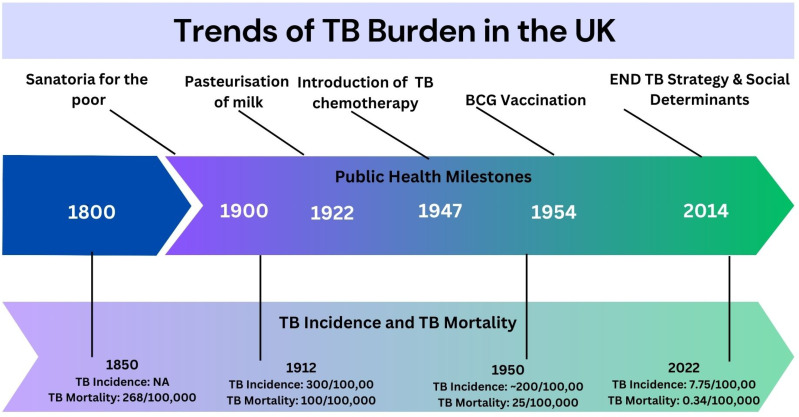
Historical trends in TB mortality and TB incidence in the United Kingdom. Source of data: references [12,13].

Firstly, many countries, like the United Kingdom, which currently has a low TB burden, had high rates of TB incidence and TB mortality in the past. The estimated mortality from respiratory TB in the UK was 268 per 100,000 in 1851 [13], and 1 in every four deaths in the UK was attributable to TB [12]. Secondly, the decline in TB incidence and mortality in the UK occurred much before public health measures or the introduction of chemotherapy or vaccination, as illustrated in Fig 1 [12,13]. The mortality from respiratory TB had declined by nearly 50% to 139 per 100,000 in 1891 [13]. In the UK, TB incidence derived from notifications was around 300 per 100,000 in 1913, while the TB death rate was 100 per 100,000 population [12]. The annual decline in TB incidence in the pre-chemotherapy era was around 3% (the same as currently seen in high TB-burden countries). By 1940, TB incidence and mortality rates were 50% of that in 1912 [12]. Later, the introduction of chemotherapy for TB led to a 10% annual decline [12]. A similar pattern of significant decline in TB burden in the pre-chemotherapy era followed by accelerated decline following chemotherapy was seen in other countries in Western Europe and the US [11].

In 1962, Thomas McKeown published a paper that considered four factors underlying the decline of TB mortality in the UK between 1850 and 1950 [13]. These factors were specific preventive or curative therapy, changes in the population’s virulence, genetic selection, and changes in the environment with improved living standards. Under the environmental changes, he considered the role of conditions of exposure, rest, and improvement in nutrition. He considered each alternative explanation and, in a deductive approach based admittedly on that of Sherlock Holmes, concluded that the decline was related to improved living standards during the period of interest, especially improved nutrition. McKeown proposed that improved nutrition with consequent improvement in host resistance to disease was a plausible explanation for the decline in TB mortality; and cited indirect evidence supporting this explanation [13]. At the time of the publication of this article, the mechanisms underlying adaptive immunity against TB and its relationship to nutrition were unknown.

Now, there is direct evidence of the historical improvements in nutrition and knowledge about the pathways through which nutrition could reduce TB incidence and mortality. Nutrition improved with the increase in the working-class real earnings in the United Kingdom (UK) post-1850. The trend between semi-logarithmic values of respiratory tuberculosis mortality is a virtual mirror image of the trend of the semi-logarithmic values of the working-class real earnings in the UK, as pointed out by Phillip D’Arcy Hart [14]. The Nobel prize-winning economist Robert Fogel uncovered direct historical evidence of increased calorie intake in the UK between 1850 and 1950 [15]. Another piece of evidence is the increase in the height of European men (including Britishers) between 1870 and 1970s by 11 cm, or more than 1 cm increase per decade, which directly reflects the effect of improved nutrition in a population [16]. Height is considered a good marker of the net nutritional status of a population and an index of the standard of living [17].

Evidence has been presented to show that the decrease in TB incidence and mortality in the UK was accompanied in the same period by improvements in nutrition. The linkage between improved nutrition and decline in TB incidence and mortality was established later by an understanding of the effect of nutrition on immune function as described below.

### Nutrition, infections, nutritionally acquired immune deficiency syndrome, and TB

The WHO Monograph reviewing the Interactions between Nutrition and Infections was a milestone in establishing the bidirectional relationship between nutritional status and infections [18]. It showed that nutritional status affects the frequency, severity, and fatality of many common infections, including tuberculosis, and infections, in turn, worsen the nutritional status [18]. The basis for this interaction was uncovered with the discovery of elements of cell-mediated and humoral components of adaptive immunity in the late 1960s and early 1970s; [19] and the later demonstrations of the multiple impairments in the physical barrier, innate immunity, and humoral and cell-mediated immunity in undernutrition [20]. The component of cell-mediated immunity mediated by T cells and macrophages was discovered to be crucial to protection from TB, and undernutrition was found to impair T cell function [21].

The natural history of TB illustrates that the role of infection is essential, but that of the immune system is crucial. In people exposed to TB infection, only 10% progress to active TB over a lifetime, reflecting the immune system’s protective effect [9]. Thus, the development of active TB reflects some immune system dysfunction. The US Surgeon General referred to malnutrition as the leading cause of acquired correctable immune system dysfunction [22], and the worldwide prevalence of malnutrition is much higher than that of HIV infection. The immune dysfunction associated with undernutrition has been called nutritionally acquired immunodeficiency syndrome(N-AIDS) by Dr. William Beisel [23], which is as relevant to TB control as HIV/AIDS [24]. This N-AIDS is the leading risk factor for TB incidence globally and in many high TB-burden countries [24,25]. The definition of undernutrition is itself the subject of discussion and research. While the WHO uses anthropometric indices (e.g., BMI < 18.5 kg/m2 as a threshold for undernutrition in adults), nutritionists are suggesting a higher cutoff (e.g., BMI < 20 kg/m^2^) along with weight loss-based criteria and measures of muscle mass [26].

## Adequate balanced food: a vaccine with a unique product profile

In the current context, Adequate Balanced Food (ABF) that is *adequate* in energy and protein (depending on the age, weight, and level of activity), *balanced* with a variety of nutrient-dense whole grains, legumes, fruits, and vegetables, healthy fats with nuts and seeds, and animal source foods(milk, eggs, poultry, fish), qualifies as a vaccine against the Acid-Fast Bacilli (AFB) causing TB [4,24] Table 1 shows that ABF is a vaccine with a product profile that other vaccines will find hard to match.

**Table 1:** The unique product profile of the Adequate Balanced Food vaccine (ABF) for tuberculosis and more.

1. Adequate Balanced Food can be preventive and therapeutic in patients with tuberculosis, both for short- and long-term adverse outcomes. 2. Adequate Balanced Food is a vaccine that can be administered orally and does not require a cold chain for transport. 3. Adequate Balanced Food is a polyvalent vaccine that may protect not only against many infectious diseases but also against some non-communicable diseases and improve public health indicators. 4. Adequate Balanced Food is safe for children and pregnant and lactating mothers. 5. Adequate Balanced Food is a vaccine that can be grown on farms and safely dispensed over the counter. 6. Adequate Balanced Food as a vaccine has no issue of Intellectual Property Rights, but it is an issue of human rights. 7. Adequate Balanced Food is a vaccine that is guaranteed high compliance, giving recipients a sense of well-being. 8. Adequate Balanced Food is a vaccine with intergenerational effects. 9. Adequate Balanced Food is a vaccine that promotes health and growth and improves cognition and productivity. 10. Adequate Balanced Food can attenuate or potentiate the effects of existing vaccines for TB and other diseases.

The following 10 features of the product profile of this vaccine, along with supporting evidence, are offered for this proposition.

1. ABF can be preventive and therapeutic in PwTB, both for short- and long-term adverse outcomes.

Adequate, balanced food may serve as a vaccine for disease prevention, as well as a vaccine for prevention of death (POD) and a vaccine for prevention of recurrence (POR). It can prevent death during TB treatment by addressing the risk factor of moderate to severe undernutrition, [27], which is highly prevalent in PwTB in high-burden countries [28–30]. In the RATIONS trial in which nearly half of PwTB were severely underweight, a 5% weight gain over baseline in the first 2 months reduced the hazard of death by over 60% [31]. On the contrary, in a cohort of patients with drug-susceptible TB not provided nutritional support, most patients had a static or decreasing BMI in the first 2 months, and this was associated with a 5-fold risk of mortality [32]. In a systematic review, being underweight also increased the risk of post-TB treatment mortality, with 2-year mortality being 14.8% in underweight patients compared to 5.6% in not underweight patients [33]. PwTB who are underweight at the start of treatment and have inadequate weight gain have nearly 2 times the risk of recurrent TB [34].

2. ABF is a vaccine that can be administered orally with no requirement for a cold chain

An ideal vaccine would be one that is not only effective but also available for oral administration and without an onerous requirement for a cold chain for its storage and distribution. Breastmilk is the earliest balanced food vaccine that humans receive whose value in disease prevention is well-known. Later in life, freshly cooked food, balanced in terms of macronutrients and micronutrients, sustains life and prevents disease. Frozen and processed foods have specific production, storage, and transport requirements and are not considered in this argument.

3. ABF is a polyvalent vaccine protective against other infectious diseases, some noncommunicable diseases (NCDs) like diabetes, and other public health problems

Undernutrition makes many infections more frequent and severe, which nutritional supplementation with a balanced diet can reduce [18]. These include the common lower respiratory infections, diarrheal infections, tuberculosis, and measles, all nutritionally sensitive diseases [18,35]. The impact of undernutrition on common infections in childhood is devastating. In 2022, there were nearly 4.9 million deaths of under-five children (or more than 13,000/day), and nearly half of these were due to the deadly combination of malnutrition and infection [36]. In field studies in Guatemala during 1959–1964, nutritional supplementation alone was provided in a village without improved medical care or sanitation [37]. A lower prevalence of infections was seen in the village with supplementary feeding compared to a treatment village where excellent medical care and sanitation improvements were provided, a significant difference of 6.6 illnesses/child compared to 18.7 illnesses/child in 4 years [37]. Mortality due to measles was eliminated [38].

Undernutrition early in life has consequences for health in the neonatal, infancy, and preschool years and lifelong health. Maternal undernutrition leads to fetal undernutrition and intrauterine growth restriction marked by low birth weight. Fetal undernutrition has been linked, based on epidemiological and animal studies, to a higher risk of NCDs like obesity and diabetes [39,40], hypertension [41], cardiovascular disease [42], and chronic kidney disease [41,43] in later life. In Low-middle-income countries (LMICs), which are experiencing a rapid increase in NCDs, low birth weight is common [44] and may contribute to their burden beyond those due to unhealthy diets and sedentary lifestyles [45]. Unhealthy diets are also often related to food insecurity with a lack of access, availability, and affordability of adequate balanced food and consumption of energy-dense processed foods high in sugar, saturated fats, and sodium and poor in nutrients [46]. The pathway to increased risk of non-communicable diseases is mainly due to structural and functional adaptations to intrauterine stress and epigenetic modifications that can be heritable [47]. For example, children with low birth weight have reduced islet cell mass and number of beta cells [48]. This, along with low stature and low lean mass, contributes to low metabolic capacity, which, when stressed with higher metabolic load due to a high glycemic diet and sedentary behaviour [49], can result in the phenotype of diabetes seen in India -earlier onset, at lower body mass index levels, increased incidence even in normal BMI, and lower insulin secretion than seen in the West [49–51].

There has been a recent resurgence of interest in the linkage of undernutrition with diabetes in low-middle income countries. A phenotype of diabetes prevalent in individuals with low BMI (<19 kg/m^2^) with a distinctive metabolic profile characterised by insulin deficiency without evidence of any autoimmunity has been reported [52]. This was formally classified as malnutrition-related diabetes mellitus by WHO in 1985, but later dropped from the classification [53]. This phenotype has now been termed as Low-BMI diabetes or type 5 diabetes as suggested recently by the International Diabetes Federation (IDF), and is estimated to affect 20–25 million adults worldwide in Asia and Africa [54]. It has been hypothesised that this phenotype has origins in fetal undernutrition that continued in childhood and adults. Undernutrition can impaired pancreatic development and endocrine function [54,55]. So fetal undernutrition followed either by energy surplus in later life or chronic energy and protein deficiency could lead to risk of diabetes.

LBW also increases the risk of stunting and cognitive delay in childhood. [56] Childhood stunting is associated with 5–7% income losses; any measures to address it will likely give an annual 12% return on investment [57].

The effect of nutritional supplementation in early life on the risk of NCDs is a work in progress, with some cohorts reporting significant reductions in risk factors [58] while others have not [59]. However, there is compelling epidemiological and experimental evidence for the development origins of adult health and disease (DOHaD). We should make efforts to prevent NCDs by following a life-course approach starting at conception rather than in adolescence and adulthood. The first ‘intrauterine’ vaccine a mother should administer to the child should be adequate balanced food during pregnancy.

One of the most compelling demonstrations of the effect of nutrition on public health was the Wartime Food Policy adopted in Great Britain during the Second World War [60]. The food policy ensured that all citizens had access to a diet meeting their physiological requirements as far as possible, irrespective of their income. This was made possible by several measures involving home production, food imports, food rationing according to needs, subsidies on staple foods, and adequate wages [60]. The needs of expectant mothers, children, adolescents, heavy workers, and ‘invalids’ were especially attended to. The citizens’ diets saw a significant rise in consumption of milk (+28%) and vegetables (+34%), with a significant drop in meat consumption (-21%) compared to the pre-war period. Despite the general conditions of the war and medical care that could have led to the deterioration of public health indices, “The rates of infantile, neonatal, and maternal mortality and the stillbirth rate reached the lowest levels ever. The incidence of anaemia declined, the growth rate and the condition of the teeth of school-children were improved, and the general state of nutrition of the population as a whole was up to or above pre-war standards.” [60] This food policy benefitted the working class the most and eliminated the nearly 14–20% differentials in caloric and protein intake compared to the middle class of the pre-war period [61].

4. ABF is safe for children and pregnant and lactating mothers

This vaccine is safe for all population groups. Children and pregnant and lactating mothers have additional demands for adequate, balanced food as an essential requirement.

5. ABF is a vaccine that can be grown on farms and safely dispensed over the counter.

The production of regular vaccines requires ‘mass production.’ However, this vaccine can be produced by the ‘masses’ in communities and countries by optimal use of their natural and human resources. The production of this vaccine does not depend on high technology, nor does its consumption need a doctor to prescribe and a pharmacist to dispense it.

6. ABF: no issue of Intellectual Property Rights (IPRs), but an issue of human rights

Over the millennia, farmers have evolved hundreds of varieties of food crops for different conditions. In the interests of small farmers and communities in low-middle-income countries, it is essential not to compromise the food sovereignty of communities and nations through agricultural products that involve IPR. The right to food is recognized in the Universal Declaration of Human Rights and the International Covenant on Economic, Social, and Cultural Rights.

7. ABF is a vaccine that guarantees high compliance, giving recipients a sense of well-being. Tuberculosis, Undernutrition, and HIV are syndemics linked by poverty and food insecurity [62]. Peter Piot, the former head of UNAIDS, spoke thus of one visit to Malawi – “``I was in Malawi, and I met with a group of women living with HIV. As I always do, I asked them what their highest priority was. Their answer was clear and unanimous: food. Not care, not drugs for treatment, not relief from stigma, but food.“ [63] Food insecurity is a risk factor for mental health problems, including depression and anxiety [64,65]. Adequate balanced food is a non-pharmacologic means of increasing serotonin levels in the brain [66]. Adequate balanced food is one preventive and therapeutic vaccine where there is unlikely to be any vaccine hesitancy.
8. ABF is a vaccine with intergenerational effects:

“It must be borne in mind that the diet of a given generation may affect several generations.” [67]. The effects of a poor diet can be seen across generations as offspring of underweight mothers are likely to be underweight themselves and, in turn, likelier to give birth to underweight babies themselves [68]. This can perpetuate the cycle of undernutrition and, due to the associated adverse outcomes of health and disease, perpetuate the cycle of poverty. In recent years, with the emergence of the science of epigenetics, it is now known that genes are not set in stone, and environmental influences, including undernutrition, can alter the expression of genes rather than changing them [69]. These epigenetic marks can explain the long-term impact of environmental influences (both positive and negative) and are heritable and transmissible to future generations. [69] This has reframed the nature vs. nurture into an artificial dichotomy. Nurture can affect nature, and both are important.

9. ABF promotes health and growth and improves cognition and productivity

Adequate, balanced food is fundamental to producing a state of health. Adequate energy, protein, and micronutrient intake ensures physical growth, cognitive development, and productivity. Evidence from the child cohorts in Guatemala shows that nutritional supplementation given in early childhood significantly impacted educational attainment and economic productivity in later life [70,71]. Undernutrition is causing a large-scale invisible brain drain in families, communities, and countries [72]. Adequate nutrition and socially provided basic needs like education, sanitation, and primary healthcare could be the most effective way of empowering the poor to escape poverty.

10. ABF can attenuate or potentiate the effects of existing vaccines for TB and other diseases

Vaccines stimulate humoral and cell-mediated immune responses to pathogens or their products.

An adequate immune response depends on the supply of all essential nutrients. Nutritional status can affect responses to vaccination. In general, there is evidence of adequate response in patients with undernutrition to protein vaccines (e.g., Diphtheria-Pertussis-Tetanus), subunit vaccines (e.g., hepatitis B), and polysaccharide vaccines (e.g., pneumococcal and meningococcal) [73,74]. However, the titres reached by underweight recipients were lower [75]. Similarly, in a recent study of Ugandan children, only 75% of infants had a protective antibody titre at 1 year, and undernutrition was a predictor of poor response [75]. In the case of live vaccines, there is evidence from old studies that the delayed-type hypersensitivity (DTH) response to BCG is reduced in those with mild-moderate protein-calorie malnutrition [74,76] and more so with severe undernutrition [77]. In the case of killed vaccines for typhoid, undernutrition was associated with a lower titre [78,79]. In a study of Gambian children, nutritional supplementation was associated with a higher titre of antibodies to Salmonella protein than a control group [78]. In the recent trial of M72/ASO1_E,_ the vaccine’s efficacy compared to the placebo was 42.1% in those with a BMI < 25 kg/m2 compared to 81.8% in those with a BMI > 25 kg/m2. However, the difference was not statistically significant [5]. Taken together, improvements in nutrition may reduce the frequency and severity of infections by their effect on the immune system and improving responses to vaccination.

## Concluding reflections

The description of adequate balanced food as an effective, therapeutic, preventive, oral, polyvalent, safe, growth promoting, happiness-inducing vaccine that can serve as an adjuvant for other vaccines, can be manufactured without IPR issues in farms, and dispensed over the counter may seem hyperbolic to some. Yet continuing to neglect undernutrition in the population - the major driver of TB incidence in populations in low, middle-income countries or the management of moderate and severe undernutrition in patients is unlikely to result in our achieving the end TB goals of reducing TB incidence or TB mortality [80].

The protective efficacy of food baskets in the RATIONS trial or Red Cross food parcels for POWs in World War II living in conditions of overcrowding and stress is derived from the efficacy of the most effective system/vaccine for preventing TB we already have. This is the protection conferred by the immune system, and for an intracellular organism like Mycobacterium tuberculosis, the cell-mediated immunity (CMI). The lifetime risk of progression of latent TB infection to active TB is 5–15% [81]. If the immune system is considered a vaccine, this would translate into an 85–95% efficacy as a post-exposure vaccine. When cellular immunological health is affected by the widespread prevalence of factors such as undernutrition, HIV, diabetes, smoking, or immunosuppressive factors, we witness epidemics of TB. Of this, undernutrition contributes to the largest proportion of new cases of TB*. Providing adequate, balanced food is like feeding the immune system and restoring its health.* Nutritional status is one of the key determinants of a well-functioning immune system. Quality protein intake is one of the key deficiencies in the diet in high TB-burden countries, impacting undernutrition and its consequences from stunting to cellular immunodeficiency [82]. These populations in LMICs in Africa and Asia heavily depend on cereals and starchy staples for their protein intake, with low intakes of quality protein sources like pulses, legumes, and animal-source foods [83]. E.g., in India, according to the recent national family health survey data, less than half of adults consumed any pulses or milk daily, and daily consumption of other animal-source foods is seen in less than ten percent [84].

The conventional strategies for TB prevention are either limited in their efficacy in terms of population group and duration of protection (BCG vaccination) or logistically daunting (roll out of TB prevention treatment in high TB burden countries which requires implementation of sequential steps of identifying populations at risk, ruling out disease, testing for TB infection and administering TB preventive treatment). We should continue researching more effective vaccines for TB but also be mindful of a few caveats. The earliest of these vaccines may be available only in 2028. Secondly, their efficacy will also depend on the state of immunocompetence of the recipients. These vaccines may well protect people with better health but may likely fail to demonstrate high efficacy in those with immunosuppression due to undernutrition, HIV, or diabetes. Finally, we may have challenges accessing these vaccines in LMICs due to cost and IPRs. So, while we search for effective vaccines or roll out implementation, it may be prudent to simultaneously work on improving population health that might reduce TB incidence here and now and improve vaccine efficacy when made available. However, this improvement of population health is a public health intervention rather than a TB-specific intervention that will have multiple benefits beyond TB control and will require multisectoral and multistakeholder actions and could be one focus for multisectoral actions to achieve the end TB goals and other SDG goals.

For too long, adequate balanced food and nutrition have fallen in the cracks of the false dichotomies created between adult and child nutrition (where nutrition is given importance only in children under five and pregnant and lactating women), or micronutrient and macronutrient nutrition (where micronutrients have received focus at the expense of addressing energy and protein deficits). Finally, nutrition has faced exclusion in the way that we classify health problems as communicable and non-communicable (while nutrition affects them both). It has acquired an urgency because of two immediate threats – climate change and conflicts in the world that are destabilizing food security for the world’s vulnerable, regardless of where they live. Food inflation is the component of inflation that is hurting the most. In its latest report, the Food and Agriculture Organisation states that 2.33 billion worldwide are moderately to severely food insecure [81]. We face increasing disparities in access to adequate, balanced food amongst the most vulnerable. In 2022, according to FAO estimates in low-income countries, 71.5% could not afford a healthy diet, whereas the proportion for high-income countries was 6.3% [85].

TB control will have to move beyond the metaphor of war on a bacillus to also advocate for the improvement of the health of people by addressing the basic biological and social needs of people everywhere. Dr. Martin Luther King Jr. articulated some of these in his acceptance speech for the Nobel Prize for Peace in 1964, “I have the audacity to believe that people everywhere can have three meals a day for their bodies, education, and culture for their minds and dignity, equality and freedom for their spirits.”
